# Supplementary material for: A large Late Miocene cetotheriid (Cetacea, Mysticeti) from the Netherlands clarifies the status of Tranatocetidae
Source: PeerJ. 2019 Feb 13;7:e6426. doi: 10.7717/peerj.6426 (PMC6377596; doi:10.7717/peerj.6426)
Supplement: Table S1 — R, reworked. [file peerj-07-6426-s001.pdf]

Table S1. Palynomorphs assemblage associated with NMR9991-16680. R, reworked

**Dinoflagellate cysts**	**Total abundance**
Achomosphaera andalousiensis	5
Achomosphaera spp.	1
Amiculosphaera umbracula	1
Barssidinium graminosum	38
Bitectatodinium tepikiense	1
Cribroperidinium spp.	1
Dinocyst indet.	1
Enneadocysta pectiniformis	1R
Glaphyrocysta spp.	1R
Habibacysta tectata	9
Hystrichosphaeropsis obscura	2
Lejeunecysta spp.	1
Lingulodinium machaerophorum	10
Melitasphaeridium choanophorum	2
Operculodinium centrocarpum	3
Operculodinium piaseckii	1
Operculodinium spp.	2
Polysphaeridium spp.	1
Reticulatosphaera actinocoronata	4
Selenopemphix armageddonensis	2
Selenopemphix brevispinosa	2
Selenopemphix dionaeacysta	5
Selenopemphix spp.	1
Spiniferites spp.	62
Tectatodinium pellitum	2
Trinovantedinium spp.	1
**Sporomorphs**	
Bisaccates	12
Sporomorphs (excl. bisaccates)	5
**Miscellaneous fossils**	
Foraminifera	17
Paralecaniella spp.	1
